# Supplementary material for: Morphological and Molecular Investigation of Non-Simulium damnosum Black Flies in Cameroon Using Nuclear ITS 2 and Mitochondrial Cox 1 Genes
Source: Insects. 2025 May 28;16(6):572. doi: 10.3390/insects16060572 (PMC12193153; doi:10.3390/insects16060572)
Supplement: Supplementary file 1 [file insects-16-00572-s001.zip › Supplementary file S1.pdf]

Supplementary file S1. Sample details and GenBank accession numbers of individual species used for DNA sequence analysis with ITS2 in Fig. 4 and for photographs in Fig. 2

Species	Location, collection date, collector	Code	Accession numbers	Photo in Fig. 2
S. dentulosum	Type A: Menchum falls (2018, PK)<br>Type B: Bambui (2018, PK)<br>Type C: Mawong river (2018, PK),	MR82<br>3d4<br>2d4	MZ475117<br>MZ475116<br>MZ475115	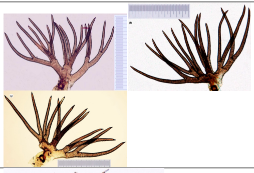
***S. medusaeforme* f. Pomeroy**	Mawong river (2018, PK)<br>Menchum falls (2018, PK)<br>Bambui (2018, PK)<br>Menchum falls (2018, PK)	MR101<br>3M3<br>3M2<br>2M1	MZ475100<br>PQ849256<br>MZ475101<br>MZ475099	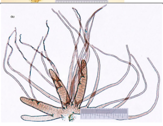
S. medusaeforme f. hargreavesi	Vina du Sud falls (2019, PK, DE)	VC6		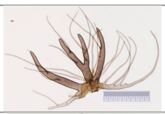
***S. undescribed* 1**	Menchum falls (2018, PK)<br>Menchum falls (2028, PK)	2V1<br>2V5	MZ475113<br>PQ849257	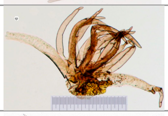
***S. undescribed* 2**	Aladji Marafat (2020, PK)	AM1		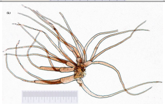
S. adersi	Mawong river (2018, PK)	MR113	OQ382883	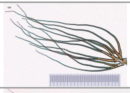
S. alcocki	Mawong river (2018, PK)<br>Menchum falls (2028, PK)	MR71<br>MR112	MZ475119<br>PQ849252	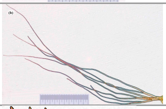
S. cervicornutum	Mawong river (2018, PK)<br>Menchum falls (2018, PK)<br>Aladji Marafat (2020, PK)	MR434<br>2C1<br>AM4	MZ475105<br>MZ475106<br>MZ475107	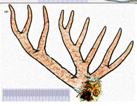
S. unicornutum	Mawong river (2028, PK)	MR12<br>MR13	PQ849249<br>PQ849250	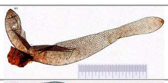
S. schoutedeni	Mawong river (2018, PK)	MR51	MZ475108	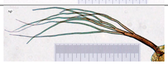
S. hirsutum	Mawong river (2018, PK)	MR31	OQ378916	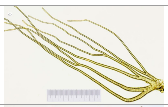
S. kantangae	Menchum falls (2018, PK)<br>Mawong river (2028, PK)	2k2<br>MR213	MZ475103<br>MZ475104	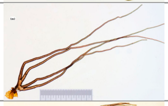
S. nigritarsis	Vina du Sud falls (2020, PK)<br>Vina du Nord (2020, PK)<br>Vina du Nord (2020, PK)	VC3,<br>Bi1,<br>Bi2	PQ849253<br>PQ849255<br>PQ849254	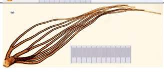
S. vorax	Vina du Sud falls, (2020, PK & DE)	VC1	MZ475112	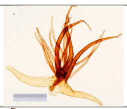
S. ruficorne	Mawong river (2018, PK)	MR92	PQ849251	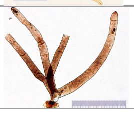

All samples were from pupae, collected by hand and fixed in alcohol (see materials & methods).
