# Supplementary material for: Morphological and Molecular Investigation of Non-Simulium damnosum Black Flies in Cameroon Using Nuclear ITS 2 and Mitochondrial Cox 1 Genes
Source: Insects. 2025 May 28;16(6):572. doi: 10.3390/insects16060572 (PMC12193153; doi:10.3390/insects16060572)
Supplement: Supplementary file 1 [file insects-16-00572-s001.zip › Supplementary file S2.pdf]

Supplementary file S2: Species count and percentage distribution across locations

SN	Location	Species Name	Count	Percentage
1	Touboro	*S. bovis*	8	47.1%
		S. cervicornutum	1	5.9%
		S. vorax	8	47.1%
2	Karna Manga	*S. kenyae*	2	100.0%
3	Aladji Marafat	*S. medusaeforme* f. Pomeroy	30	83.33%
		S. undescribed 2	1	2.78%
		S. nigritarsis	4	11.11%
		S. medusaeforme f. *hargreavesi*	1	2.78%
4	Vina du Sud	*S. adersi*	1	12.50%
		S. medusaeforme f. *hargreavesi*	3	37.50%
		S. nigritarsis	2	25.0%
		S. vorax	2	25.0%
5	Mayo Djouroum	*S. medusaeforme* f. Pomeroy	5	20.8%
		S. vorax	19	79.2%
6	Mawong River	*S. adersi*	6	1.2%
		S. alcocki	12	2.4%
		S. cervicornutum	308	62.5%
		S. dentulosum	12	2.4%
		S. hirsutum	50	10.1%
		S. katangae	34	6.9%
		S. kenyae	3	0.6%
		S. undescribed 1	7	1.4%
		S. ruficorne	5	1.0%
		S. medusaeforme f. *hargreavesi*	6	1.2%
		S. schoutedeni	3	0.6%
		S. unicornutum	55	11.2%
7	Menchum Falls	*S. alcocki*	3	1.8%
		S. cervicornutum	100	59.5%
		S. medusaeforme f. *hargreavesi*	1	0.6%
		S. dentulosum	10	6.0%
		S. hirsutum	8	4.8%
		S. katangae	8	4.8%
		S. medusaeforme f. Pomeroy	1	0.6%
		S. undescribed 1	23	13.7%
		S. unicornutum	12	7.1%
8	Bambui	*S. alcocki*	22	5.7%
		S. cervicornutum	198	51.4%
		S. dentulosum	25	6.5%
		S. katangae	110	28.6%
		S. undescribed 1	13	3.4%
		S. unicornutum	10	2.6%
9	Mbanga	//	*0*	0.0%
10 - 13	Nkongsamba	*S. cervicornutum*	2	15.4%
		S. dentulosum	1	7.7%
		S. katangae	2	15.4%
		S. medusaeforme f. Pomeroy	4	30.8%
		S. ruficorne	4	30.8%
14	Soramboum	*S. alcocki*	1	3.0%
		S. medusaeforme f. *hargreavesi*	32	97.0%
