# Supplementary material for: Morphological and Molecular Investigation of Non-Simulium damnosum Black Flies in Cameroon Using Nuclear ITS 2 and Mitochondrial Cox 1 Genes
Source: Insects. 2025 May 28;16(6):572. doi: 10.3390/insects16060572 (PMC12193153; doi:10.3390/insects16060572)
Supplement: Supplementary file 1 [file insects-16-00572-s001.zip › Supplementary file S3.pdf]

Supplementary file S3: Species used to align samples in this study using the ITS2 gene.

**Species names**	**GenBank accessions numbers**	**Countries of origin**	**References**	**publication status**
S. ruficorne	JQ673503	Reunion	Gomard,Y. et *al*., 2012	Unpublished
S. ruficorne	JQ673505	Reunion	Gomard,Y. et *al*., 2012	Unpublished
S. ruficorne	JQ673507	Reunion	Gomard,Y. et *al*., 2012	Unpublished
S. ruficorne	KY421709	Morocco	Gomard,Y. et *al*., 2017	Unpublished
S. ruficorne	KY421710	Morocco	Gomard,Y. et *al*., 2017	Unpublished
S. ruficorne	KY421702	Morocco	Gomard,Y. et *al*., 2017	Unpublished
S. balcanicum	KF990278	Turkey	Inci, A. et *al*., 2013	Unpublished
S. turgaicum	KF990276	Turkey	Inci, A. et *al*., 2016	Unpublished
S. lineatum	KF990280	Germany	Inci, A. et *al*., 2013	Unpublished
S. paraequinum	KC414865	Iran	Khazeni,A. et *al*., 2012	Unpublished
S. equinum	OM721760	Russia	Vaulin,O.V. et *al*., 2022	Published
S.(Eusimulium) latipes	MZ475111	Germany	Kamtsap, P. et *al*., 2021	Unpublished
S. pseudequinum	OR482416	Turkey	Kaya,S. et *al*., 2023	Unpublished
S. pseudequinum	OR482419	Turkey	Kaya,S. et *al*., 2023	Unpublished
S. pseudequinum	OR482423	Turkey	Kaya,S. et *al*., 2023	Unpublished
S. pseudequinum	OQ473250	Spain	Ruiz-Arrondo,I., 2023	Published
S. squamosum	AY625925	Cameroon	Krueger,A. et *al*., 2004	Published
S. triplex	JQ673511	Reunion	Gomard,Y. et *al*., 2012	Unpublished
S.(Odagmia) ornatum	MZ475114	Germany	Kamtsap, P. et *al*., 2021	Unpublished
S.(Odagmia) ornatum	EU429855	England	Day,J.C. and Goodall,T.I., 2008	Unpublished
